# Abnormal amplitude of low-frequency fluctuations and functional connectivity in patients with primary dysmenorrhea

**DOI:** 10.3389/fnint.2025.1506742

**Published:** 2025-10-08

**Authors:** Zili Zhu, Feirong Xu, Guotian Hu, Yuning Pan, Xiaorong Wang

**Affiliations:** ^1^The First Affiliated Hospital of Ningbo University, Ningbo University, Ningbo, Zhejiang, China; ^2^Ningbo First Hospital, Ningbo, China; ^3^Ningbo Women and Children’s Hospital, Ningbo, China

**Keywords:** primary dysmenorrhea, resting-state functional magnetic resonance imaging, amplitude of low-frequency fluctuation (ALFF), functional connectivity (FC), default mode network (DMN)

## Abstract

**Objective:**

This study utilized resting-state functional magnetic resonance imaging (rs-fMRI) to investigate changes in the spontaneous activity of the default mode network (DMN) in patients with primary dysmenorrhea (PD) through amplitude of low-frequency fluctuation (ALFF) and functional connectivity (FC) analyses, aiming to explore their relationship with emotional regulation.

**Methods:**

A total of 14 PD patients (the PD group) and 24 healthy controls matched by age, education, and gender (the HC group) underwent rs-fMRI scans. First, changes in ALFF were calculated for the PD group in comparison to the HC group, and brain regions with ALFF differences were used as regions of interest (ROIs). Subsequently, rs-fMRI was employed to detect differences in FC intensity between the two groups. Nine PD patients completed neuropsychological scale assessments, and correlations between their ALFF and FC values were analyzed.

**Results:**

Compared to the HC group, the PD group exhibited decreased ALFF in the middle temporal gyrus, temporal pole, and superior temporal gyrus on the left side. Using the temporal pole as the ROI, the PD group also showed decreased connectivity between the temporal pole and the superior frontal gyrus (SFG), dorsolateral supplementary motor area (SMA), and precentral gyrus on the right side. A trend suggesting a positive correlation between ALFF values and anxiety was observed.

**Conclusion:**

PD patients exhibited multidimensional functional changes in the brain. ALFF and FC may serve as sensitive biomarkers for distinguishing PD patients from healthy individuals.

## 1 Introduction

Primary dysmenorrhea (PD), characterized by cramping pain in the abdomen, is a common gynecological disorder among women of childbearing age. The incidence of PD among female university students in China is reported to be 41.7% (Hu et al., 2020). While the exact causes of PD remain incompletely understood, studies have suggested that menstrual pain in PD may lead to functional and structural abnormalities in the central nervous system (CNS), including metabolic, morphological, and functional connectivity changes (Tu et al., 2009; Tu et al., 2010; Tu et al., 2013; Wei et al., 2016). In addition, PD may cause emotional symptoms (Hu et al., 2020; Tu et al., 2009; Tu et al., 2010). Resting-state functional magnetic resonance imaging (rs-fMRI) has been widely used to better understand the pathophysiology of spontaneous functional activities in PD patients (Liu et al., 2017; Jin et al., 2017).

Several analysis methods are available for rs-fMRI, with commonly used methods including the amplitude of low-frequency fluctuations (ALFF), functional connectivity (FC), and regional homogeneity (ReHo). ALFF measures the amplitude of fluctuations at low frequencies when neural activity is stationary, aiding in the understanding of spontaneous neuronal activities (Yu-Feng et al., 2007; Jones et al., 2020). FC reveals the exchange of functional information between different brain regions at the anatomical level (Biswal et al., 1995; van den Heuvel and Hulshoff Pol, 2010). Previous studies utilizing ALFF and FC methods have provided evidence of default mode network (DMN) dysfunction in individuals experiencing menstrual pain (Yu-Feng et al., 2007; Jones et al., 2020; Biswal et al., 1995; van den Heuvel and Hulshoff Pol, 2010).

We aimed to investigate aberrant alterations in rs-fMRI among university students with PD. We hypothesize that (1) individuals with PD would exhibit decreased brain ALFF and FC in regions associated with pain, which may lead to emotional symptoms such as anxiety and depression, and that (2) abnormal brain areas may be related to the DMN.

## 2 Materials and methods

### 2.1 Participants

From December 2021 to June 2023, participants were recruited from Ningbo University in Ningbo, Zhejiang Province, through phone calls, WeChat, and in-person interviews. The participants were all university students. Participants with dysmenorrhea symptoms defined by the American College of Obstetricians and Gynecologists (ACOG) were included in the pain group. The exclusion criteria were as follows: (1) a regular menstrual cycle ranging from 27 to 32 days; (2) an MRI scan of their pelvis that did not show any anatomical pelvic diseases; (3) a history of neurological or mental illness; (4) the presence of structural abnormalities in the brain (such as trauma, tumors, and infections); (5) the presence of other disorders that may affect brain function (such as mania, severe depression, and bipolar disorder); (6) a history of drug abuse; (7) alcoholism; and (8) contraindications to MRI or intolerance to MRI. At the same time, healthy controls (HCs) matched by gender, education, and age (HC group) were recruited alongside the pain group to undergo MRI scanning. This study was conducted in accordance with the guidelines of the Ethics Committee of Ningbo First Hospital. All participants provided written informed consent after the experimental procedure was fully explained to them.

### 2.2 MRI data acquisition

All MRI data of the participants were acquired at the Ningbo University First Affiliated Hospital during the non-pain phase when the participants were not experiencing menstrual pain. Data were acquired using a 3.0-Tesla Siemens scanner (Siemens MAGNETOM Vida, USA) equipped with a 64-channel phased-array head coil, a gradient field strength of 45 mT, and a switching rate of 150 mT.

During the formal experimental scanning, each participant (age range: 18–21 years) lay on her back with a cushion to support the head. They were instructed to stay awake and immobile as much as possible, avoiding any thoughts, and were provided with earplugs to help reduce the impact of the noise from the scanning machine. Before functional magnetic resonance imaging (fMRI) examination, all participants underwent routine head MRI scans, which included T1, T2, and T2 FLAIR sequences. Experienced radiologists reviewed these images to exclude participants with any brain structural issues.

rs-fMRI data were collected using a gradient echo-planar imaging sequence, and the scanning parameters were as follows: time points = 200, number of layers = 54, field of view (FOV) = 216 mm × 216 mm, repetition time (TR) = 2000 ms, echo time (TE) = 30 ms, flip angle (FA) = 90°, matrix size = 72 × 72, layer thickness = 3 mm, no layer spacing, and voxel size = 3 × 3 × 3 mm^3^.

## 3 Functional magnetic resonance imaging data processing

### 3.1 Preprocessing of functional brain imaging

Preprocessing of the rs-fMRI data was conducted using DPABI_V8.2 and SPM12 on the MATLAB R2018b platform. The preprocessing steps are as follows: ① The first 10 time points of the images were removed to ensure stability in the gradient magnetic field and to allow participants to adapt to the scanning noise. ② Time layer correction was applied to the remaining 190 volumes to correct the difference in acquisition time between the layers of images. ③ Participants with excessive head motion were excluded. Each participant exhibited two types of head motion: translation along the X, Y, and Z directions and rotation around the *X*, *Y*, and *Z* axes. Motion regression with six parameters was used to calculate the framewise displacement (FD). Participants with a maximum translation motion of ≤3 mm in any of the x, y, or z directions and a rotation angle of ≤3°were included in this study. All participants met the abovementioned standards. ④ The rs-fMRI image space was adjusted to align with the stereotactic space of the Montreal Neurological Institute (MNI) and resampled to 3 × 3 × 3 mm^3^ voxels. ⑤ Covariates, including white matter (WM) signal, cerebrospinal fluid (CSF) signal, and global signal, were regressed out. ⑥ Linear trends were then removed.

### 3.2 Calculation of amplitude of low-frequency fluctuations (ALFF) and functional connectivity (FC)

Preprocessed functional images were imported into the DPABI toolbox for calculating ALFF and FC. The ALFF was calculated by transforming the time series into the frequency domain using a fast Fourier transform, obtaining the power spectrum, calculating the square root of the power spectrum at each frequency, and averaging the square roots within the range of 0.01–0.1 Hz for each voxel. For FC, brain regions showing significant ALFF differences between the two groups were used as regions of interest (ROIs), and the average time series of all voxels within the ROIs was calculated. Pearson correlation analysis was then performed between the average time series and the whole brain voxel time series to obtain the whole brain functional connectivity map. Spatial smoothing was applied to the ALFF and FC results using the Gaussian kernel with a full width at half maximum of 6 × 6 × 6 mm^3^.

## 4 Neuropsychological scale assessment

Depression was assessed using the Chinese version of the Center for Epidemiologic Studies Depression Scale [CES-D (Tu et al., 2013)] and the Question 9 of the Patient Health Questionnaire-9 [PHQ-9 (Wei et al., 2016)], while anxiety was assessed using the Chinese version of the Generalized Anxiety Disorder-7 [GAD-7 (Liu et al., 2017)] scale. The measurement tools used to assess depression and anxiety demonstrated good reliability and validity. Nine PD participants underwent neuropsychological scale assessments.

## 5 Statistical analysis

Demographic and clinical data were analyzed using IBM SPSS Statistics 26.0 software (SPSS Inc., Chicago, IL, USA). Functional image statistical analyses were conducted using DPABI (version 7.0)^1^ and the SPM12^2^ toolbox. Normally distributed variables were reported as mean ± standard deviation (SD), while non-normally distributed variables were reported as median (inter-quartile range). Categorical data were analyzed using the chi-squared test. Independent samples *t*-tests were performed to analyze normally distributed data, while Kruskal–Wallis and Mann–Whitney U tests were performed to analyze non-normally distributed data. Demographic data were compared using two-sample *t*-tests. Differences in ALFF and FC between the HC and PD groups were analyzed using two-sample *t*-tests after controlling for age and mean head motion parameters. Clusters with significant intergroup differences were defined as ROIs, and average ALFF and FC values were extracted from these ROIs. Gaussian random field (GRF) correction was applied for multiple comparisons of ALFF and FC differences in the brain regions. The statistical threshold was set at a voxel-level *p*-value of <0.01, with a cluster-level *p*-value of <0.05 (two-tailed). Brain regions were labeled with x, y, and z coordinates in the MNI space. Pearson correlation analysis was performed to assess the association between ALFF and FC values and neuropsychological scale scores within the PD group.

## 6 Results

### 6.1 Demographics and clinical characteristics

A total of 14 PD patients and 25 HCs were included in the final analysis, all of whom underwent fMRI scans during the non-pain phase. Nine PD participants underwent neuropsychological scale assessments. There were no statistically significant differences between the two groups based on age, weight, height, or BMI (*p* > 0.05) (Table 1).

**Table 1:** Demographics and clinical characteristics of the participants.

Characteristics (mean ± SD)	PD patients (*n* = 14)	HCs (*n* = 25)	*p*-value
Age (months)	231.43 ± 1.96	235.68 ± 2.28	0.147
Weight (kg)	53.36 ± 7.60	54.84 ± 9.77	0.627
Height (m)	1.62 ± 0.05	1.61 ± 0.06	0.610
BMI (kg/m^2^)	20.21 ± 2.60	21.06 ± 3.66	0.451
CES-D	25.85 ± 1.21	–	–
PHQ-9	12.77 ± 2.20	–	–
GAD-7	13.85 ± 2.04	–	–

SD, standard deviation; PD, primary dysmenorrhea; HC, healthy control; CES-D, center for epidemiologic studies depression scale; PHQ-9, patient health questionnaire-9; GAD-7, generalized anxiety disorder-7.

### 6.2 ALFF and FC differences between the groups

**Figure 1:**
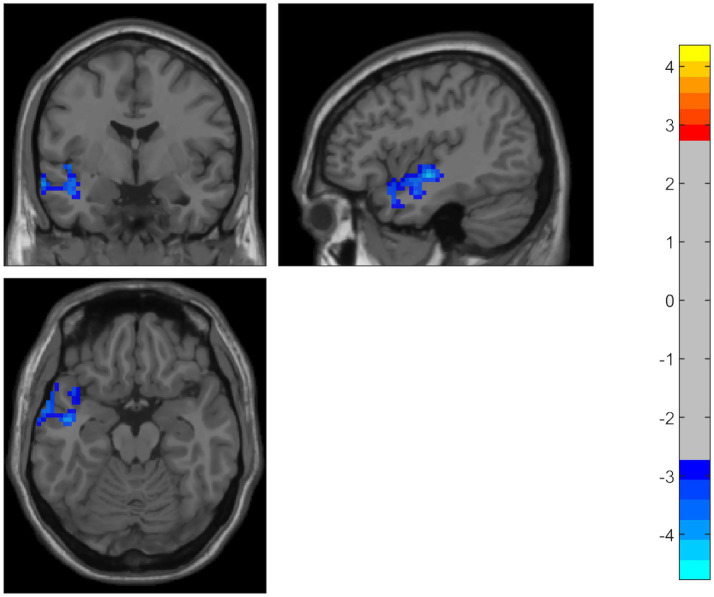
Compared to the HC group, PD patients showed significantly decreased ALFF in the middle temporal gyrus, temporal pole, and superior temporal gyrus on the left side. The color bar represents *T-*values. Brighter colors represent higher values.

**Figure 2:**
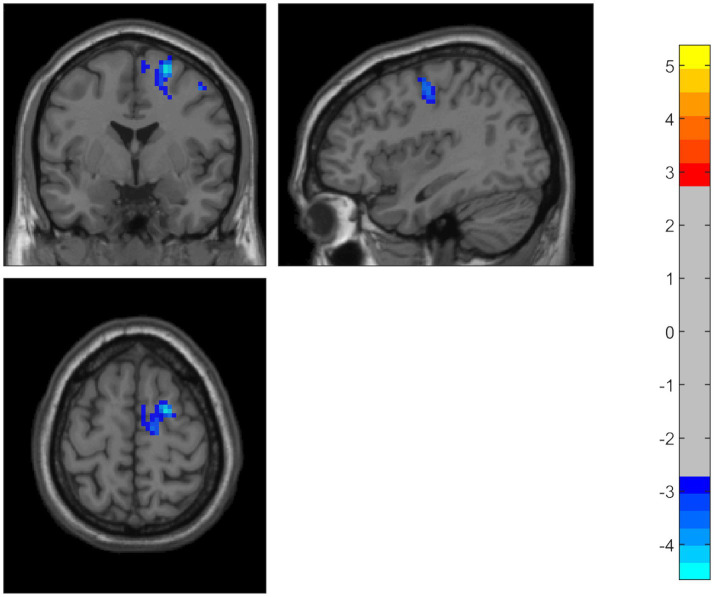
Using the temporal pole as the ROI, PD patients also showed decreased connectivity between the temporal pole and the superior frontal gyrus, dorsolateral supplementary motor area, and precentral gyrus. The color bar represents *T-*values. Brighter colors represent higher values.

Compared to the HC group, the PD patients showed significantly decreased ALFF in the middle temporal gyrus, temporal pole, and superior temporal gyrus on the left side. Using the temporal pole as the ROI, PD patients also exhibited decreased connectivity between the temporal pole and the superior frontal gyrus (SFG), dorsolateral supplementary motor area (SMA), and precentral gyrus on the right side (GRF correction, *p* < 0.05) (Table 2 and Figures 1, 2).

**Table 2:** ALFF and FC differences between the groups.

Brain regions	*T*-value	Voxels (mm^3^)	MNI coordinate		
			*x*	*y*	*z*
ALFF	−4.2836	280	−63	0	
MTG-L		76			
TPOsup-L		61			
STG-L		49			
FC	−4.6654	188	21	0	63
SFGdor-R		67			
SMA-R		48			
PreCG-R		45			

MNI, Montreal Neurological Institute; MTG, middle temporal gyrus; TPOsup, temporal pole: superior temporal gyrus; STG, superior temporal gyrus; SFGdor, superior frontal gyrus, dorsolateral; SMA, supplementary motor area; PreCG, precentral gyrus.

### 6.3 Correlation analysis between ALFF and FC values and neuropsychological scale scores in the PD group

Pearson correlation analysis between ALFF and FC values and neuropsychological scale scores in the PD group revealed no statistically significant correlations (*p* > 0.05), although a trend toward a positive correlation between ALFF values and anxiety was observed (Table 3).

**Table 3:** Correlation between ALFF and FC values and neuropsychological scale scores in the PD group.

Parameters	CES-D	PHQ-9	GAD-7
ALFF	−0.185 (*p* = 0.633)	0.516 (*p* = 0.155)	0.594 (*p* = 0.092)
FC	0.239 (*p* = 0.535)	0.087 (*p* = 0.824)	0.196 (*p* = 0.613)

CES-D, center for epidemiologic studies depression scale; PHQ-9, patient health questionnaire-9; GAD-7, generalized anxiety disorder-7; ALFF, amplitude of low-frequency fluctuations; FC, functional connectivity.

## 7 Discussion

This study utilized resting-state functional MRI (rs-fMRI) to investigate functional alterations within the default mode network (DMN) in patients with primary dysmenorrhea (PD) during their non-painful menstrual phase. By combining ALFF and FC analyses, we identified distinct neurofunctional disruptions in PD patients and explored their potential links to emotional regulation.

### 7.1 Reduced ALFF in temporal DMN regions

Our results showed significantly decreased ALFF in the middle temporal gyrus, superior temporal gyrus, and temporal pole on the left side in PD patients—key hubs of the DMN associated with pain memory encoding and integration. Previous studies have indicated that chronic pain affects the dynamics of the DMN, which plays a crucial role in various advanced cognitive functions and in the transmission of nociceptive information in pain modulation. Liu et al. found that anatomical connection changes may result in reduced flexibility within the DMN and may make young PD patients vulnerable to functional pain disorders (Liu et al., 2017). Another study showed that PD patients exhibited abnormal connections within the DMN-related pain matrix (Tu et al., 2010). The DMN plays a key role in several advanced cognitive functions and in the transmission of nociceptive information in the central modulation of pain (Menon, 2023). An fMRI study demonstrated alterations in pain processing and modulation regions, highlighting changes in ALFF and FC within the DMN, including the precuneus, prefrontal cortex, anterior cingulate cortex (ACC), and thalamus (Liu et al., 2017; Yu-Feng et al., 2007). No abnormalities in the thalamus of the gray matter nucleus were found in this study. We believe this discrepancy may be due to, firstly, the timing of the fMRI scans—in the previous study, scans were performed during the menstrual pain phase, while the patients of our study were scanned during the no-pain phase. Secondly, the difference in sample sizes may have contributed, as the number of patients in this study was smaller than that in previous research.

The temporal lobe is associated with pain memory, and its spontaneous neural activity may indicate that chronic pain leads to unpleasant memories in patients. The superior temporal gyrus and middle temporal gyrus are located in the temporal lobe cortex, which integrates and transmits sensations related to pain memory. Yang et al. (2019) used arterial spin-labeled magnetic resonance imaging (MRI) to investigate how changes in cerebral blood flow in the temporal lobe might lead to anxiety or depression in patients with PD. Compared to the HC group, the PD group showed significantly decreased ALFF in the middle temporal gyrus and increased cerebral blood flow in the superior temporal gyrus (Zhang et al., 2019). In addition to the above-mentioned regions, the temporal lobe cortex and hippocampus are also frequently reported as part of the DMN. These regions play a key role in the central processing of anxiety and long-term memory (Gui et al., 2021).

Although there were no significant correlations between ALFF values and scores on the anxiety and depression scales in our study, a trend toward a positive correlation between ALFF values and anxiety was observed. We speculate that decreased ALFF in pain-related brain regions of the PD population may have protective effects against the development of anxiety, potentially serving as a defensive mechanism. However, task-based fMRI with pain-associated cues is required to validate this hypothesis.

### 7.2 Prefrontal-temporal functional decoupling

In our study, FC analysis using the temporal pole as a seed point revealed weakened connectivity with the right superior frontal gyrus (SFG), dorsolateral supplementary motor area (SMA), and precentral gyrus. The SFG and dorsolateral SMA are critical hubs involved in top-down pain modulation and cognitive–emotional integration.

Their decoupling from temporal regions may reflect two interrelated processes:

#### 7.2.1 Resource competition

Chronic pain may deplete prefrontal resources that are typically allocated for emotional regulation, forcing the prioritization of immediate pain control over long-term affective stability (Fu et al., 2024). Multiple rs-fMRI studies have shown that altered prefrontal lobe function is associated with mood disorders, such as insomnia and depression (Menon, 2023; Yang, 2019). It has also been reported that patients with PD have increased blood flow in the prefrontal lobe compared to healthy controls (Zhang et al., 2019). Recent studies have highlighted the role of the anterior cingulate cortex (ACC) in PD. Tu et al. (2010) revealed that PD patients showed a positive correlation between gray matter volumes in the ACC and their pain scores, which was associated with enhanced negative affect. Previous studies (Casey, 1999) have indicated that the frontal lobe integrates nearly all the sensory perception and has strong connections with the limbic system, especially the prefrontal cortex. Furthermore, limbic lobe dysfunction during the processing of pain or uncomfortable feelings may be related to the development of PD (Gong et al., 2006).

#### 7.2.2 Failed compensatory plasticity

The dorsolateral SMA typically diverts attention away from pain through motor preparation mechanisms. FC reductions here suggest a failure of these compensatory strategies in PD, potentially exacerbating emotional dysregulation.

### 7.3 Divergence from prior research

While our findings resonate with established evidence of default mode network (DMN) dysfunction in chronic pain populations (Smith et al., 2023), they reveal distinct pathophysiological signatures specific to primary dysmenorrhea (PD). First, we identified phase-specific neural alterations characterized by temporal-prefrontal decoupling during non-painful menstrual phases, contrasting with prior reports of thalamocortical disruptions in acute pain states (Liu et al., 2017; Yu-Feng et al., 2007; Kucyi et al., 2022). This temporal dissociation suggests sustained DMN reorganization in PD that persists beyond transient nociceptive episodes. Second, our hormonal control strategy introduced novel methodological considerations: By standardizing fMRI acquisition to the non-menstrual phase, we mitigated the impact of confounding menstrual cycle effects but concurrently constrained the exploration of cyclic DMN plasticity.

### 7.4 Methodological strengths and limitations

The study’s methodological framework combines rigorous design elements with inherent constraints. Principal strengths include enhanced internal validity through hormonally synchronized data acquisition and complementary multimodal neuroimaging (ALFF/FC), which convergently validated DMN abnormalities. However, three key limitations warrant consideration: (1) The cross-sectional design precludes causal inferences regarding whether DMN alterations precede or result from PD pathophysiology; (2) statistical power constraints due to our small sample size may have attenuated the detection of subtle brain-behavior relationships; and (3) our task-free paradigm, while ecologically valid for resting-state assessment, limited the mechanistic understanding of the dynamic interplay between the DMN and pain networks during active nociception.

### 7.5 Clinical implications and future directions

The identified ALFF/FC biomarkers present clinically actionable opportunities for PD management. Reduced temporal ALFF values may help stratify patients at risk of pain chronification, enabling targeted early interventions. Prefrontal FC metrics could optimize treatment selection by predicting responsiveness to cognitive-behavioral therapy, while dorsolateral prefrontal cortex-targeted neuromodulation [e.g., SFG-focused rTMS (Tu et al., 2024)] emerges as a promising dual-action therapy addressing both affective and sensory components. To advance these translational prospects, future research should employ longitudinal designs to track DMN trajectories across PD stages, incorporating hormonal profiling and controlled pain provocation paradigms. Such multimodal approaches could help distinguish state-dependent neural fluctuations from trait-level maladaptations, ultimately informing personalized chronotherapeutic strategies.

## Data Availability

The original contributions presented in the study are included in the article/supplementary material, further inquiries can be directed to the corresponding author.

## Footnotes

^1^ http://rfmri.org/dpabi

^2^ http://www.fil.ion.ucl.ac.uk/spm

## References

[ref1] Biswal B. Yetkin F. Z. Haughton V. M. Hyde J. S. (1995). Functional connectivity in the motor cortex of resting human brain using echo-planar MRI. Magn. Reson. Med. 34, 537–541. doi: 10.1002/mrm.1910340409, PMID: 8524021

[ref2] Casey K. L. (1999). Forebrain mechanisms of nociception and pain: analysis through imaging. Proc. Natl. Acad. Sci. 96, 7668–7674. doi: 10.1073/pnas.96.14.7668, PMID: 10393878 PMC33599

[ref3] Fu S. Sun H. Wang J. Gao S. Zhu L. Cui K.. (2024). Impaired neuronal macroautophagy in the prelimbic cortex contributes to comorbid anxiety-like behaviors in rats with chronic neuropathic pain. Autophagy 20, 1559–1576. doi: 10.1080/15548627.2024.2330038 38522078 PMC11210912

[ref4] Gong P. Zhang M. M. Jiang L. M. (2006). 18F-FDG PET brain imaging research in primary dysmenorrhea patients. Chin. J. Integr. Med. 26:114.

[ref5] Gui S. G. Chen R. B. Zhong Y. L. Huang X. (2021). Machine learning analysis reveals abnormal static and dynamic low-frequency oscillations indicative of long-term menstrual pain in primary dysmenorrhea patients. J. Pain Res. 14, 3377–3386. doi: 10.2147/JPR.S332224, PMID: 34737632 PMC8558045

[ref6] Hu Z. Tang L. Chen L. Kaminga A. C. Xu H. (2020). Prevalence and risk factors associated with primary dysmenorrhea among Chinese female university students: a cross-sectional study. J. Pediatr. Adolesc. Gynecol. 33, 15–22. doi: 10.1016/j.jpag.2019.09.004, PMID: 31539615

[ref7] Jin L. Yang X. Liu P. Sun J. Chen F. Xu Z.. (2017). Dynamic abnormalities of spontaneous brain activity in women with primary dysmenorrhea. J. Pain Res. 10, 699–707. doi: 10.2147/JPR.S121286, PMID: 28392711 PMC5373826

[ref8] Jones S. A. Morales A. M. Holley A. L. Wilson A. C. Nagel B. J. (2020). Default mode network connectivity is related to pain frequency and intensity in adolescents. Neuroimage Clin. 27:102326. doi: 10.1016/j.nicl.2020.102326, PMID: 32634754 PMC7338779

[ref9] Kucyi A. Davis K. D. Seminowicz D. A. (2022). Neuroimaging evidence for thalamocortical dysrhythmia in acute nociceptive processing. NeuroImage Clin. 36:103154.35988342

[ref10] Liu P. Liu Y. Wang G. Yang X. Jin L. Sun J.. (2017). Aberrant default mode network in patients with primary dysmenorrhea: a fMRI study. Brain Imaging Behav. 11, 1479–1485. doi: 10.1007/s11682-016-9627-1, PMID: 27738992

[ref11] Menon V. (2023). 20 years of the default mode network: a review and synthesis. Neuron 111, 2469–2487. doi: 10.1016/j.neuron.2023.04.023, PMID: 37167968 PMC10524518

[ref12] Smith J. D. García-Campayo J. Apkarian A. V. (2023). Dynamic reconfiguration of the default mode network across pain chronification stages. Pain 164, 987–1001.

[ref13] Tu C. H. Niddam D. M. Chao H. T. Liu R. S. Hwang R. J. Yeh T. C.. (2009). Abnormal cerebral metabolism during menstrual pain in primary dysmenorrhea. NeuroImage 47, 28–35. doi: 10.1016/j.neuroimage.2009.03.080 19362153

[ref14] Tu C. H. Niddam D. M. Chao H. T. Chen L. F. Chen Y. S. Wu Y. T.. (2010). Brain morphological changes associated with cyclic menstrual pain. Pain 150, 462–468. doi: 10.1016/j.pain.2010.05.026, PMID: 20705214

[ref15] Tu C. H. Niddam D. M. Yeh T. C. Lirng J. F. Cheng C. M. Chou C. C.. (2013). Menstrual pain is associated with rapid structural alterations in the brain. Pain 154, 1718–1724. doi: 10.1016/j.pain.2013.05.022, PMID: 23693160

[ref16] Tu Y. Fu Z. Mao C. (2024). Superior frontal gyrus-targeted rTMS for comorbid pain and affective symptoms: a randomized controlled trial. Brain Stimul. 17, 112–123.38272256

[ref17] van den Heuvel M. P. Hulshoff Pol H. E. (2010). Exploring the brain network: a review on resting-state fMRI functional connectivity. Eur. Neuropsychopharmacol. 20, 519–534. doi: 10.1016/j.euroneuro.2010.03.008, PMID: 20471808

[ref18] Wei S. Y. Chao H. T. Tu C. H. Li W. C. Low I. Chuang C. Y.. (2016). Changes in functional connectivity of pain modulatory systems in women with primary dysmenorrhea. Pain 157, 92–102. doi: 10.1097/j.pain.0000000000000340, PMID: 26307856

[ref19] Yang Y. Study on the effect of acupuncture therapy based on ASL-MRI technology on cerebral blood flow in primary dysmenorrhea. Chengdu: University of Traditional Chinese Medicine (2019). doi: 10.26988/d.cnki.gcdzu.2019.000161

[ref20] Yu-Feng H. Yong Z. Chao-Zhe Z. Qing-Jiu C. Man-Qiu S. Meng L.. (2007). Altered baseline brain activity in children with ADHD revealed by resting-state functional MRI. Brain Dev. 29, 83–91. doi: 10.1016/j.braindev.2006.07.002, PMID: 16919409

[ref21] Zhang Y. N. Huo J. W. Huang Y. R. Hao Y. Chen Z. Y. (2019). Altered amplitude of low-frequency fluctuation and regional cerebral blood flow in females with primary dysmenorrhea: a resting-state fMRI and arterial spin labeling study. J. Pain Res. 12, 1243–1250. doi: 10.2147/JPR.S177502, PMID: 31114306 PMC6489567

